# Is a High Homocysteine Level Related to Thrombosis?

**DOI:** 10.5505/tjh.2012.80106

**Published:** 2012-12-05

**Authors:** Şinasi Özsoylu

**Affiliations:** 1 Retired Prof of Pediatrics, Hematology, Hepatology, Ankara, Turkey; 2 Honorary Fellow of American Academy of Pediatrics; 3 Honorary Member of American Pediatric Society

**To the Editor,**

The study by Eğin and Akar [1] entitled, First observation of MTHFR 678C-A (Ala 222 Ala) single nucleotide polymorphism, in the recent issue of the Journal (2012; 29: 204-5) requires questioning its importance to thrombosis in the light of Dayal et al.’s findings in a mouse model [2]. I used to think that high homocysteine level important in thrombosis, whereas currently I question such knowledge. As such, I think the authors must show the functional effect of their observation in blood coagulation and its relationship to the thermolabile variant of MTHFR ?.
